# Total Knee Arthroplasty Considerations in Rheumatoid Arthritis

**DOI:** 10.1155/2013/185340

**Published:** 2013-09-16

**Authors:** Jonathan R. Danoff, Garrett Moss, Barthelemy Liabaud, Jeffrey A. Geller

**Affiliations:** Center for Hip & Knee Replacement, New York-Presbyterian Hospital at Columbia University, New York, NY 10032, USA

## Abstract

The definitive treatment for advanced joint destruction in the late stages of rheumatoid arthritis can be successfully treated with total joint arthroplasty. Total knee arthroplasty has been shown to be a well-proven modality that can provide pain relief and restoration of mobility for those with debilitating knee arthritis. It is important for rheumatologists and orthopedic surgeons alike to share an understanding of the special considerations that must be addressed in this unique population of patients to ensure success in the immediate perioperative and postoperative periods including specific modalities to maximize success.

## 1. Introduction

Over the past thirty years, major advances have been realized in the understanding of the pathogenesis and treatment of rheumatoid arthritis (RA). As an immune mediated process, all joints are affected, as synovitis leads to destruction of cartilage, which may ultimately result in bone loss and joint deformity. Joint contractures, fixed flexion and valgus deformities, and ligamentous laxity are especially evident in large joints, complicating treatments. With the advent of highly effective biologic therapies, fewer individuals with rheumatoid arthritis suffer this end-stage joint destruction [1]. Despite this success, approximately 20–25% of afflicted individuals develop advanced arthritis in their joints, with the knee being one of the most commonly affected joints contributing to patient pain and overall disability [2, 3]. Total knee arthroplasty (TKA) has proven to be a highly successful treatment for advanced rheumatoid arthritis. The orthopedic surgeon must pay special attention to the unique challenges presented by this population of patients during preoperative, intraoperative, and postoperative planning in order to maximize successful outcome and quality of life for these patients. 

## 2. Preoperative Considerations

Rheumatoid arthritis is a systemic disease, which creates a unique set of challenges and considerations when treating patients afflicted with this disease. Care is often delivered by a variety of specialty physicians, including rheumatologists and orthopedic surgeons. Preoperative communication is vital among these providers to maximize outcomes. 

Surgeons and anesthesiologists alike must be aware of the increased risk to the cervical spine as 80% of patients have atlantoaxial instability [4]. The cervical spine also is at elevated risk of basilar invagination and subaxial instability, and thus preoperative radiographic investigation via flexion and extension images should be obtained, as intubation for surgery can often require hyperextension of the patient's neck, and should be avoided should instability be present [5]. When atlantoaxial instability exists, the patient should be referred to a spine surgeon for stabilization prior to any elective joint arthroplasty procedure.

It is well established that individuals with end-stage joint destruction, secondary to rheumatoid arthritis, tend to be an average of ten years younger compared to those with osteoarthritis (OA) [5]. Despite the decreasing overall rates of TKA for patients aged 40–59, the overall number of procedures has increased due to an expanding population [1]. This may create special demands on a device that has a limited life span. As populations continue to age and more individuals choose arthroplasty, the overall number of complications, including infection and aseptic loosening secondary to polyethylene wear, will continue to rise, making revision surgery ever more challenging in an already complicated population. Despite the challenges, midterm and long-term outcomes have demonstrated the success of TKA in improving quality of life. Ranawat et al. presented his series of midterm outcomes at 6.1 years of 93 cemented TKAs (17 OA, 73 RA). Good or excellent results were seen in 97.7% of the knees, and authors predicted a 96% survivorship at 10 years [6]. Gill et al. demonstrated good-to-excellent long-term outcomes at 9.9 years for patients undergoing cemented TKA younger than 55 years, comparing 29 knees with RA to 37 knees with OA [7]. Authors concluded that the “good” outcomes in 10% of their patients were due to the multiply affected joints in rheumatoid arthritis. This led Lee and Choi to suggest that these patients should not be evaluated by the functionality of each joint individually, but rather on their overall physiologic status instead of numeric age when considering surgical intervention [5].

The difficulty to control the polyarticular nature of the disease is further challenging physicians. Tanaka et al. demonstrated that the most commonly affected joints are the wrist, metacarpophalangeal joint, and proximal interphalangeal joints, although the majority of the pain and disability is secondary to shoulder, knee, and hip pathology [3]. When combined together in the same patient, the overall prognosis is exponentially worsened, and often hip and knee synovitis and arthritis are coexistent [8]. It is accepted that in those patients with ipsilaterally affected hip and knee joints with end-stage arthritis, total hip arthroplasty (THA) should be performed first, prior to TKA [5]. Controversy exists on whether upper or lower extremity pathology should be addressed first. Clement et al. argue that stresses placed on the upper extremity during therapy for TKA may further damage previously repaired joints [4]. Conversely, Chmell et al. believe that upper extremity impairment such as severe wrist pain due to RA may impair lower extremity rehabilitation, and thus wrist fusion should be considered prior to TKA. It is our practice to evaluate all afflicted joints prior to arthroplasty to determine the influence of other joints on rehabilitation and outcome.

## 3. Perioperative Medication Management

Rheumatoid arthritis is generally treated using a combination of medications including nonsteroidal anti-inflammatory drugs (NSAIDs), glucocorticoids, and disease-modifying antirheumatic drugs (DMARDs). NSAIDs have also played a smaller role in symptomatic management, but these COX-1 and nonselective COX inhibitors can be associated with increased bleeding risk and should be withheld 1 week prior to surgery [5]. In our practice we prescribe COX-2 inhibitors (Celecoxib) postoperatively, as other studies have suggested that the slight increase in bleeding risk does not significantly increase the need for transfusion [9]. 

Glucocorticoids, on the other hand, can be used to treat flares of disease and as a maintenance medication, and its use in the perioperative period must be specifically tailored to the patient requirements [5]. It must be considered that patients who have received a prolonged treatment course of steroids may be prone to secondary adrenal insufficiency secondary to the chronic suppression of corticotrophin-releasing hormones from the hypothalamus. These patients require stress doses perioperatively of 50–100 mg of hydrocortisone or 10–15 mg of methylprednisone intravenously with an immediate taper to prevent an Addisonian crisis [10]. Additionally, chronic glucocorticoid use is associated with poor bone quality, compromise of the immune system, and impaired wound healing. Studies have demonstrated that rheumatoid arthritis patients undergoing joint replacement who take chronic steroids have increased rates of joint infection, reaching 2.5–3 times that of the general population [10–12]. Thus, meticulous sterile technique, careful intraoperative skin handling, and tight closure are essential.

The most influential intervention to delay or prevent end-stage joint destruction has been DMARDs. Included in this category are methotrexate, leflunomide, sulfasalazine, azathioprine, hydroxychloroquine, tumor necrosis factor alpha (TNF-*α*) inhibitors (etanercept, infliximab, adalimumab), and interleukin-1 (IL-1) inhibitors (anakinra), as listed in Table 1. These biologic agents help control the various cytokines produced in this disease that help limit proliferation of fibroblasts, destruction of bone and cartilage, and progression of disease. Additionally, it is well established that TNF-*α*, a proinflammatory cytokine is produced by synovial cells and chondrocytes. This led Toki et al. to conclude that synovectomy and chondrocyte resection that occurs with TKA help to further improve patient outcome, complementing the action of the DMARDs systemically [13]. However, these medications are associated with an increased risk of opportunistic infections. Despite this, it is crucial to avoid reflexive continuation or cease of the use of DMARDs in the perioperative period, as this may be associated with an inflammatory flare in the smaller joints resulting in stiffness, added pain, and swelling, thus compromising the patient's ability to rehabilitate [10, 14, 15]. Here, communication between the orthopedic surgeon and rheumatologist is essential to achieve equilibrium between risk reduction for infection and disease suppression.

Few studies have been published that demonstrate the safety of these medications perioperatively. Grennan et al. published their findings from a prospectively randomized clinical study of 388 cases of rheumatoid arthritis patients undergoing joint arthroplasty (see Table 2) [15]. They found that at one-year follow-up, the group that continued to take methotrexate throughout the perioperative period had significantly better outcome, compared to those who temporarily held the medication or who used a different DMARD or corticosteroid. Sreekumar et al. reexamined the same population of patients at 10 years from surgery and found that continuing methotrexate in the long term was not associated with any additional cases of deep bone infection [16]. While the majority of patients should continue to take methotrexate perioperatively, in patients with renal dysfunction, it is prudent to hold this medication 1 week preoperatively and 1-2 weeks postoperatively. This is because the postoperative period may cause added stress to the kidneys worsening underlying renal insufficiency and propagating methotrexate toxicity [10]. Hayashi et al. investigated the TNF-*α* inhibiting DMARDs in their prospective cohort of 45 cases of RA in patients requiring large total joint arthroplasty, which included 33 TKA (see Table 2) [17]. At one-year follow-up, they were able to conclude that TNF-*α* inhibitors are safe and efficacious perioperatively when held for a short period of time.

As there exist limited prospective studies on this topic, we have delineated our preferred regimen (see Table 1) derived from the available literature. 

## 4. Intraoperative and Technical Considerations

**Figure 1:**
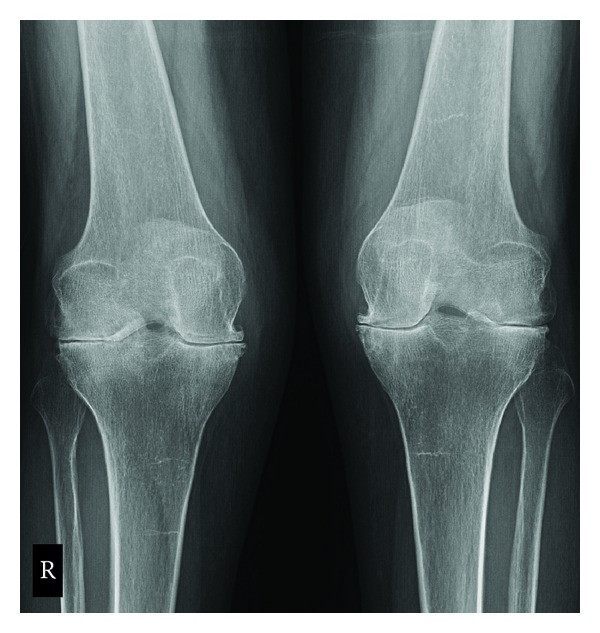
Posteroanterior radiographic view of bilateral knees demonstrating advanced arthritis. Note the valgus alignment to both legs. RA and OA radiographs differ in that RA radiographs will show periarticular erosions and osteopenia, whereas periarticular osteophytes, subchondral sclerosis, and joint space narrowing are more common in OA.

**Figure 2:**
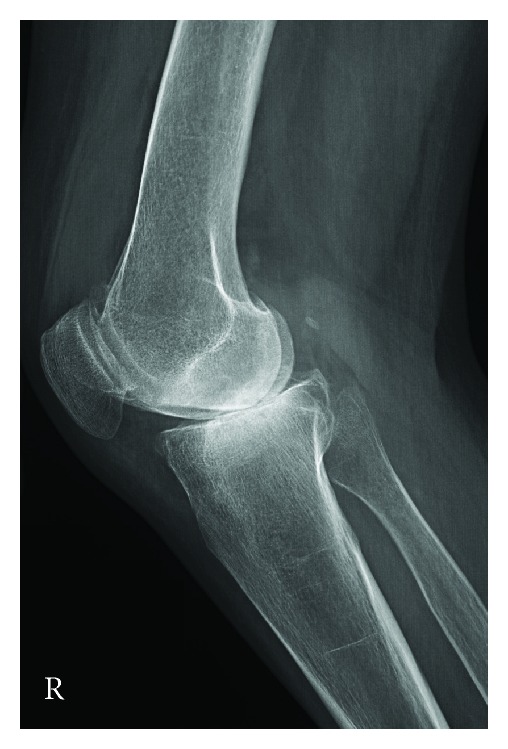
Lateral radiograph of the right knee. A small effusion is present as well.

**Figure 3:**
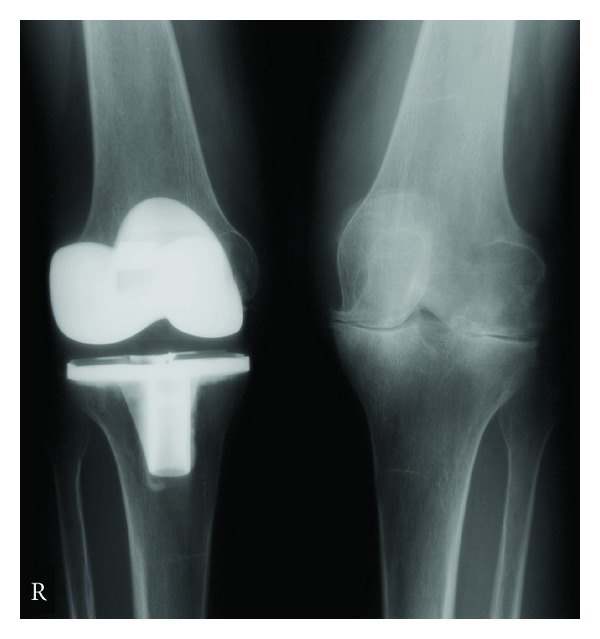
The same patient from Figures 1–3, now 22 months after right TKA was performed. The PA radiographs are seen with improved joint alignment in the right knee. The patient is doing well, walking without pain in the right knee, and is now being considered for a left TKA.

**Figure 4:**
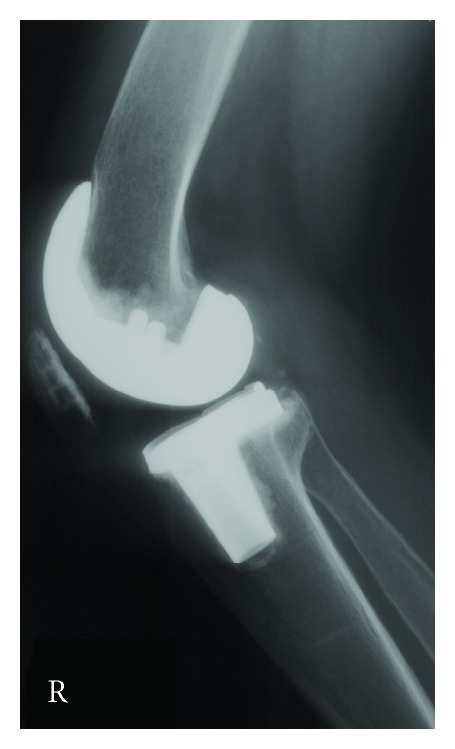
Lateral view of the right knee 22 months after TKA.

In addition to the preoperative and perioperative considerations of RA patients, there are specific technical challenges that these patients present during the TKA procedure which are not present in primary OA, as these patients are generally younger [18]. The musculoskeletal problems of RA patients include both inferior bone and soft tissue quality. The direct autoimmune nature of RA, as well as the common use of corticosteroids in these patients, results in poor subchondral bone substrate, which is needed for strong implant fixation. This resultant osteopenia is important to recognize, as this may decrease implant longevity and may also result in significant bone cysts. While small cysts can be filled during cementation, larger cysts require filling with either autologous graft from the bone cuts or allograft from morselized femoral head. Similarly, the soft tissue structures that stabilize the knee of RA patients may also be poor, often resulting in ligamentous laxity and joint deformity. This presents a special concern for the surgeon, because gross ligament instability may require increased constraint in the prosthesis, yet this increased constraint can transmit shear and rotational forces to the bone-cement interface resulting in debonding and premature failure. Fortunately, with the advanced medical management of RA, most patients with RA present with mild coronal plane deformity, though a fixed valgus deformity is sometimes present (see Figures 1 and 2). Fixed flexion contractures may also occur, thus further potentiating the overall complexity of the RA patient. Final outcomes of such a case are seen in Figures 3 and 4.

An essential component of the reconstruction involves full joint synovectomy to lessen the inflammatory process, which may continue postoperatively if not fully addressed as an inflammatory synovitis [19]. When performing a synovectomy, it is important to preserve the fatty tissue between the synovium and the anterior femur to prevent scarring and adhesion formation [12].

The use of a cruciate-retaining (CR) versus a posterior-stabilized (PS) prosthesis is still controversial. The posterior cruciate ligament (PCL) is a structure taking origin on the lateral aspect of the medial femoral condyle and inserting on the tibial plateau between the intercondylar notch and posterolateral tibial plateau on the posterior surface. This intraarticular ligament has multiple functions, but is mainly used in TKA for assisting with femoral rollback during knee flexion in order to achieve knee hyperflexion. Although excellent results have been reported with the CR prosthesis, there are some concerns that late instability due to PCL attenuation may occur with longer-term follow-up [20, 21]. Some have recommended that the PCL should be assessed intraoperatively and perform CR TKA in patients only when the PCL was present and functioning normally. Hanyu et al. assessed the PCL intraoperatively and performed CR TKA in patients only when the PCL was present and functioning normally. In their series, 10-year survivorship of the entire TKA cohort (both CR and PS) was 93%. No revisions were performed for instability in the CR group [22]. Miller et al. found a twenty-year implant survival rate of 93%, when PCL insufficiency was the endpoint [23].

## 5. Complications 

RA patients are at twice the risk of developing infection, regardless of the site, than the non-RA population [24]. By the later stage of the degenerative process, many patients have been taking several immune suppressing medications including DMARDs, glucocorticoids, and TNF-*α* inhibitors for a lengthy period of time. Even though most of these medicines are stopped before surgery, the risk of infection remains quite high with an increased risk of opportunistic infections [25, 26]. Additionally, the majority will restart their treatment postoperatively. At 5 years after primary TKA, RA patients had 3 times more infections than OA patients (4.2% compared to 1.4%) [25]. Not all drugs seem to have the same effect on the infection site; for instance, patients taking TNF-*α* inhibitors are shown to be more prone to develop superficial infection of the surgical site, but not deep bone infections [27].

Surgical wound healing is also a major concern for this group of patients. Many have a history of corticosteroid use, which is known to blunt the inflammatory phase of wound healing and to alter the remodeling of the wound; however, the dose of corticosteroids received by RA patients is not high enough to generate these issues [28, 29]. Methotrexate has also been shown to reduce wound tensile strength, but similar to corticosteroid dosages, the amount usually given to an RA patient is relatively small and usually does not influence the healing [15]. Some studies have shown more wound dehiscence in the TNF-*α* inhibitor group [29]. This is another reason why we temporarily withhold these agents in the perioperative period and as detailed in Table 1. The British Society for Rheumatology has taken this one step further and declared, “Treatment may be restarted post-operatively if there is no evidence of infection and once wound healing is satisfactory.” [30]

One area where RA patients are at lower risk for complication is deep venous thromboembolism (DVT). It is well established that lower extremity orthopedic surgery is associated with a risk of DVT in up to 50% patients undergoing large joint replacement. Deadly pulmonary embolism occurs in one to six percent of patients not taking DVT prophylaxis medications. Previous studies have reported that the incidence of DVT in patients with RA undergoing hip or knee surgery is three to ten times less than that in patients with OA [31, 32]. In both studies, the decreased occurrence of DVT in patients with RA was attributable to a younger age of patients and the frequent use of NSAIDs with the resultant antiplatelet activity. Matta et al. in their study show that DVT risk in hospitalized RA patients is higher than the non-RA population; however, one exception occurs when patients undergo joint surgery, where RA patients had a decreased risk of developing DVT in comparison to OA patients [33]. 

In a recent meta-analysis, Ravi et al. looked at the complications following total joint replacement in RA patients [34]. They conclude that RA patients were at higher risk of infection following TKA, compared to OA patients; nonetheless, they found no difference regarding risk of revision, 90-day mortality, or venous thromboembolic events within 90 days of TKA.

## 6. Conclusions

Rheumatoid arthritis is predominantly found in small joints such as fingers and wrists; however it is well understood that the majority of the impairment of activities of daily living stems from the impairment to the large joints (knee, hip, and shoulder). Early diagnosis by primary care physicians and referral to rheumatologists for aggressive treatment of rheumatoid arthritis remain paramount. For these patients with end-stage degenerative changes in the knee, total knee arthroplasty has proven in long-term follow-up studies to be a highly successful solution for patients suffering from advanced joint destruction secondary to rheumatoid arthritis. Complications after TKA are more frequent compared to the general population and represent significant adverse events requiring revision surgery. Thus, as DMARDs continue to be widely used to decrease rates of disease progressing to end stages, a common understanding, by orthopedic surgeons and rheumatologists alike, of the proper balance and timing of medications to control the systemic illness, while minimizing adverse events perioperatively, is paramount.

## Figures and Tables

**Table 1:** Disease-modifying antirheumatic drugs (DMARDs) dosing regimen in the perioperative period. The preoperative withholding period prior to surgery is specified as well as the waiting period postoperatively prior to reinstituting the medication.

Agent	Preoperative hold	Postoperative restart
Methotrexate^∧^ [15, 16]	No hold	No hold
Leflunomide [5]	1-2 days	1-2 weeks
Sulfasalazine [5]	1 day	3 days
Hydroxychloroquine [5]	No hold	No hold
Etanercept [10, 17]	1 wk	10–14 days
Infliximab [10, 17]	End dose cycle	10–14 days
Adalimumab [10, 17]	End dose cycle	10–14 days
Anakinra [10]	1-2 days	10 days

^∧^In patients with normal renal function, methotrexate should be continued throughout the preoperative/postoperative period. If renal function is abnormal, the medication should be held 1 week prior to surgery and restarted 1-2 weeks postoperatively, after the immediate stresses of surgery have subsided.

**Table 2:** Literature review of DMARD safety in the perioperative period.

	Medication	Patients	Infections	Flares
	Methotrexate			
[15]	Continued	88	2 (2%)	0
	Held Medication^∧^	72	11 (15%)	6 (8%)
	Other DMARD/steroid	228	24 (11%)	9 (4)

	TNF-*α* Inhibitor*			
[17]	Etanercept	33	0	—
	Infliximab	22	1 (5%)	—
	Adalimumab	3	0	—

^∧^Methotrexate was held two weeks before until two weeks after surgery in this group. *Etanercept and adalimumab were held two weeks before until two weeks after surgery, while infliximab was held both four weeks prior to and after surgery.

## References

[1] Louie GH, Ward MM (2010). Changes in the rates of joint surgery among patients with rheumatoid arthritis in California, 1983–2007. *Annals of the Rheumatic Diseases*.

[2] da Silva E, Doran MF, Crowson CS, O’Fallon WM, Matteson EL (2003). Declining use of orthopedic surgery in patients with rheumatoid arthritis? Results of a long-term, population-based assessment. *Arthritis Care and Research*.

[3] Tanaka E, Saito A, Kamitsuji S (2005). Impact of shoulder, elbow, and knee joint involvement on assessment of rheumatoid arthritis using the American College of Rheumatology Core Data Set. *Arthritis Care and Research*.

[4] Clement ND, Breusch SJ, Biant LC (2012). Lower limb joint replacement in rheumatoid arthritis. *Journal of Orthopaedic Surgery and Research*.

[5] Lee JK, Choi CH (2012). Total knee arthroplasty in rheumatoid arthritis. *Knee Surgery & Related Research*.

[6] Ranawat CS, Padgett DE, Ohashi Y (1989). Total knee arthroplasty for patients younger than 55 years. *Clinical Orthopaedics and Related Research*.

[7] Gill GS, Casey Chan K, Mills DM (1997). 5- To 18-year follow-up study of cemented total knee arthroplasty for patients 55 years old or younger. *Journal of Arthroplasty*.

[8] Jacoby RK, Jayson MI, Cosh JA (1973). Onset, early stages, and prognosis of rheumatoid arthritis: a clinical study of 100 patients with 11-year follow-up. *British medical journal*.

[9] Samama CM, Bastien O, Forestier F (2002). Antiplatelet agents in the perioperative period: expert recommendations of the French Society of Anesthesiology and Intensive Care (SFAR) 2001-Summary Statement. *Canadian Journal of Anesthesia*.

[10] Howe CR, Gardner GC, Kadel NJ (2006). Perioperative medication management for the patient with rheumatoid arthritis. *Journal of the American Academy of Orthopaedic Surgeons*.

[11] Luessenhop CP, Higgins LD, Brause BD, Ranawat CS (1996). Multiple prosthetic infections after total joint arthroplasty: risk factor analysis. *Journal of Arthroplasty*.

[12] Chmell MJ, Scott RD (1999). Total knee arthroplasty in patients with rheumatoid arthritis: an overview. *Clinical Orthopaedics and Related Research*.

[13] Toki H, Momohara S, Ikari K (2008). Return of infliximab efficacy after total knee arthroplasty in a patient with rheumatoid arthritis. *Clinical Rheumatology*.

[14] Momohara S, Kawakami K, Iwamoto T (2011). Prosthetic joint infection after total hip or knee arthroplasty in rheumatoid arthritis patients treated with nonbiologic and biologic disease-modifying antirheumatic drugs. *Modern Rheumatology*.

[15] Grennan DM, Gray J, Loudon J, Fear S (2001). Methotrexate and early postoperative complications in patients with rheumatoid arthritis undergoing elective orthopaedic surgery. *Annals of the Rheumatic Diseases*.

[16] Sreekumar R, Gray J, Kay P, Grennan DM (2011). Methotrexate and post operative complications in patients with rheumatoid arthritis undergoing elective orthopaedic surgery-a ten year follow-up. *Acta Orthopaedica Belgica*.

[17] Hayashi M, Kojima T, Funahashi K (2012). Effect of total arthroplasty combined with anti-tumor necrosis factor agents in attenuating systemic disease activity in patients with rheumatoid arthritis. *Modern Rheumatology*.

[18] Poss R, Ewald FC, Thomas WH, Sledge CB (1976). Complications of total hip replacement arthroplasty in patients with rheumatoid arthritis. *Journal of Bone and Joint Surgery A*.

[19] Cooke TD, Cooke V, Richer S (1975). Localization of antigen antibody complexes in intraarticular collagenous tissues. *Annals of the New York Academy of Sciences*.

[20] Laskin RS, O’Flynn HM (1997). Total knee replacement with posterior cruciate ligament retention in rheumatoid arthritis: problems and complications. *Clinical Orthopaedics and Related Research*.

[21] Archibeck MJ, Berger RA, Barden RM (2001). Posterior cruciate ligament-retaining total knee arthroplasty in patients with rheumatoid arthritis. *Journal of Bone and Joint Surgery A*.

[22] Hanyu T, Murasawa A, Tojo T (1997). Survivorship analysis of total knee arthroplasty with the kinematic prosthesis in patients who have rheumatoid arthritis. *Journal of Arthroplasty*.

[23] Miller MD, Brown NM, Della Valle CJ, Rosenberg AG, Galante JO (2011). Posterior cruciate ligament-retaining total knee arthroplasty in patients with rheumatoid arthritis: a concise follow-up of a previous report. *Journal of Bone and Joint Surgery A*.

[24] Doran MF, Crowson CS, Pond GR, O’Fallon WM, Gabriel SE (2002). Frequency of infection in patients with rheumatoid arthritis compared with controls: a population-based study. *Arthritis and Rheumatism*.

[25] Bongartz T, Halligan CS, Osmon DR (2008). Incidence and risk factors of prosthetic joint infection after total hip or knee replacement in patients with rheumatoid arthritis. *Arthritis Care and Research*.

[26] Bernatsky S, Hudson M, Suissa S (2007). Anti-rheumatic drug use and risk of serious infections in rheumatoid arthritis. *Rheumatology*.

[27] Momohara S, Inoue E, Ikari K (2007). Risk factors for total knee arthroplasty in rheumatoid arthritis. *Modern Rheumatology*.

[28] Karukonda SR, Flynn TC, Boh EE, McBurney EI, Russo GG, Millikan LE (2000). The effects of drugs on wound healing-part II. Specific classes of drugs and their effect on healing wounds. *International Journal of Dermatology*.

[29] Barnard AR, Regan M, Burke FD, Chung KC, Wilgis EFS (2012). Wound healing with medications for rheumatoid arthritis in hand surgery. *ISRN Rheumatol*.

[30] Ledingham J, Deighton C (2005). Update on the British Society for Rheumatology guidelines for prescribing TNF*α* blockers in adults with rheumatoid arthritis (update of previous guidelines of April 2001). *Rheumatology*.

[31] van Heereveld HAEM, Laan RFJM, Van den Hoogen FHJ, De Waal Malefijt MC, Novakova IRO, Van de Putte LBA (2001). Prevention of symptomatic thrombosis with short term (low molecular weight) heparin in patients with rheumatoid arthritis after hip or knee replacement. *Annals of the Rheumatic Diseases*.

[32] Niki Y, Matsumoto H, Hakozaki A, Mochizuki T, Momohara S (2010). Rheumatoid arthritis: a risk factor for deep venous thrombosis after total knee arthroplasty? Comparative study with osteoarthritis. *Journal of Orthopaedic Science*.

[33] Matta F, Singala R, Yaekoub AY, Najjar R, Stein PD (2009). Risk of venous thromboembolism with rheumatoid arthritis. *Thrombosis and Haemostasis*.

[34] Ravi B, Escott B, Shah PS (2012). A systematic review and meta-analysis comparing complications following total joint arthroplasty for rheumatoid arthritis versus for osteoarthritis. *Arthritis and Rheumatism*.

